# Neuronal Substrates Underlying Performance Variability in Well-Trained Skillful Motor Task in Humans

**DOI:** 10.1155/2016/1245259

**Published:** 2016-07-19

**Authors:** Nobuaki Mizuguchi, Shintaro Uehara, Satoshi Hirose, Shinji Yamamoto, Eiichi Naito

**Affiliations:** ^1^Center for Information and Neural Networks (CiNet), National Institute of Information and Communications Technology (NICT), 1-4 Yamadaoka, Suita, Osaka 565-0871, Japan; ^2^Japan Society for the Promotion of Science, 5-3-1 Kojimachi, Chiyoda-ku, Tokyo 102-0083, Japan; ^3^School of Health and Sport Sciences, Osaka University of Health and Sport Sciences, 1-1 Asashirodai, Kumatori-cho, Sennan-gun, Osaka 590-0496, Japan; ^4^Graduate School of Medicine and Graduate School of Frontier Biosciences, Osaka University, 2-15 Yamadaoka, Suita, Osaka 565-0871, Japan

## Abstract

Motor performance fluctuates trial by trial even in a well-trained motor skill. Here we show neural substrates underlying such behavioral fluctuation in humans. We first scanned brain activity with functional magnetic resonance imaging while healthy participants repeatedly performed a 10 s skillful sequential finger-tapping task. Before starting the experiment, the participants had completed intensive training. We evaluated task performance per trial (number of correct sequences in 10 s) and depicted brain regions where the activity changes in association with the fluctuation of the task performance across trials. We found that the activity in a broader range of frontoparietocerebellar network, including the bilateral dorsolateral prefrontal cortex (DLPFC), anterior cingulate and anterior insular cortices, and left cerebellar hemisphere, was negatively correlated with the task performance. We further showed in another transcranial direct current stimulation (tDCS) experiment that task performance deteriorated, when we applied anodal tDCS to the right DLPFC. These results indicate that fluctuation of brain activity in the nonmotor frontoparietocerebellar network may underlie trial-by-trial performance variability even in a well-trained motor skill, and its neuromodulation with tDCS may affect the task performance.

## 1. Introduction

Human motor performance does not always end up with the same behavioral consequences, but it rather fluctuates trial by trial even in a fully acquired and well-trained motor skill [1]. This performance fluctuation seems to influence results of the competition in sports games and accidents in daily habitual activities (e.g., falling during walking).

Previous neurophysiological studies in nonhuman primates have shown that fluctuation in reaching movements is caused by the variability of preparatory neuronal firing in the premotor cortex (PM) and in the primary motor cortex (M1) [1, 2]. However, it is unlikely that the cause of the neuronal fluctuation is only restricted to these local motor areas. Rather, it may also be caused by interregional synaptic input from other nonmotor domains, because PM and M1 are known to communicate with many other frontal and parietal regions during motor preparation [3, 4].

In the present study, in order to elucidate distribution of neuronal cause for a motor-performance fluctuation, we conducted a functional magnetic resonance imaging (fMRI) experiment (fMRI experiment). In a next experiment, we applied transcranial direct current stimulation (tDCS) to the dorsolateral prefrontal cortex (DLPFC), which was identified in the fMRI experiment as one of the brain regions where the activity changes in association with a fluctuation of task performance, and tested if the tDCS affects the task performance (tDCS experiment).

In the fMRI experiment, we measured brain activity with fMRI while participants repeatedly performed a well-trained skillful sequential finger-tapping task. We identified brain regions where the activity is negatively correlated with the task performance across trials. Since we found a negative correlation between the task performance and activity in a broader range of the frontoparietocerebellar network, including the DLPFC, in the following tDCS experiment, we tried to modulate brain activity in the DLPFC and evaluated the performance change in order to examine causal relationship between the DLPFC activity and the task performance.

## 2. Materials and Methods

### 2.1. Participants

Fifteen healthy male volunteers participated in the fMRI experiment (23 ± 4 years of age; mean ± one standard deviation (SD), range 20–36 years) and 9 healthy male volunteers participated in the tDCS experiment (22 ± 1 years of age, range 21–24 years). Seven volunteers participated in both experiments. All participants were right-handed according to the Edinburgh Handedness Inventory [5]. The Ethics Committee of the National Institute of Information and Communications Technology (NICT) approved this study. All participants gave written informed consent, and the experiment was carried out according to the principles and guidelines of the Declaration of Helsinki (1975).

### 2.2. fMRI Experiment

#### 2.2.1. Motor Task

**Figure 1:**
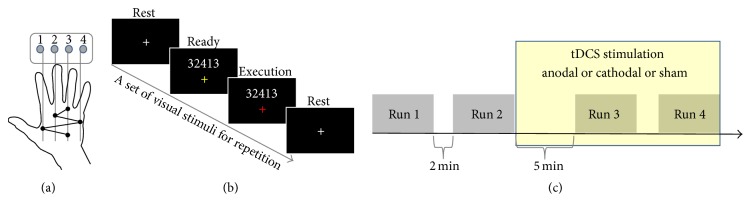
(a) A sequential finger-tapping task was performed utilizing the dominant right hand fingers. (b) Participants were required to repeatedly press a five-element sequence (sequence “32413”) as quickly and accurately as possible during a 10 s trial period. The fixation color turned yellow from white and the numeric sequence (“32413”) appeared for 3 s, indicating the ready period. A red fixation color indicated the 10 s execution period. (c) Experimental procedure of the tDCS experiment. During the 1st and 2nd runs, the participants did not receive any stimulation. The third run was begun 5 min after the start of tDCS. The participants performed the 3rd and 4th runs when receiving tDCS (anodal, cathodal, or sham) to the right dorsolateral prefrontal cortex (DLPFC).

The participants performed a sequential finger-tapping task with their dominant right fingers [6, 7]. The task was composed of five elements: ring-, middle-, little-, index-, and ring-finger tapping (pressing button), in this order (i.e., sequence “32413,” Figure 1(a)). The participants were required to repeatedly perform the same sequential tapping as quickly (many times) and accurately as possible during a 10 s trial period (see below).

#### 2.2.2. Preexperiment Training

To examine the fluctuation of the well-trained motor performance, we required the participants to intensively train the tapping task prior to the fMRI experiment (two participants who only participated in the tDCS experiment also completed this training). Training consisted of two parts: training in the laboratory and then in their own home. On the first day of training, the participants practiced a total of 50 trials of the 10 s sequential finger tapping in the laboratory. In the following days, they were asked to practice the tapping task as many times as possible in their daily life in order to feel confidence to perform the task until the date when they participated in the fMRI experiment. The interval between the laboratory training and the fMRI experiment was 9 ± 6 days (mean ± SD). We did not have direct control over the amount of practice in their home. But in order to confirm whether the task was well trained, we evaluated the performance change during the experiment in each participant (see Section 3).

#### 2.2.3. fMRI Measurement Parameters

We used a 3.0 T MRI scanner (Trio Tim, Siemens, Germany) with a head-coil to obtain T1-weighted anatomical images and functional T2^*∗*^-weighted echo-planar images (EPI: 64 × 64 matrix; pixel size = 3.0 × 3.0 mm; flip angle = 80 degrees; TE = 30 ms). Functional volumes were collected every 2 s (TR = 2,000 ms) and were comprised of 33 slices of 4.0 mm thickness with 0 mm interslice gaps, which ensured that the entire brain was within the field of view (FOV) with 192 mm × 192 mm.

#### 2.2.4. Setup for fMRI Experiment

In the scanner, the participants rested comfortably in a supine position. Their right arms were orientated parallel to their torso, and their forearms were pronated and supported by a cushion, allowing them to relax their arms. During the scan, the participants were allowed to move only their right fingers to press the buttons placed just beneath their hands (HHSC-1x4-D, Current Designs Inc., Philadelphia, USA).

Visual stimuli were projected onto a screen in the scanner. The participants viewed the stimuli via a mirror in front of their eyes. Throughout the experiment, a fixation cross was present in the center of the screen, and the participants were instructed to maintain their gaze on this point and to avoid unnecessary eye movements (Figure 1(b)). The visual stimuli were controlled by using an in-house program based on the Psychtoolbox (version 3.0; http://psychtoolbox.org/) in MATLAB (MathWorks Inc., Natick, USA). The timing of each button press was also recorded using the same program.

#### 2.2.5. Experimental Procedure

At the beginning of each trial, the numeric sequence “32413” was presented just above the fixation cross and the fixation cross turned yellow for 3 s (Figure 1(b), ready period). During this period, the participants prepared to initiate the required movement. After the ready period, the fixation cross turned red for 10 s (execution period), during which the participants repeatedly performed the sequential tapping as quickly and accurately as possible. This execution period was followed by a 9 s rest period (i.e., intertrial interval, ITI).

In the fMRI experiment, all the participants completed 15 experimental runs, with 2 min interrun intervals. Each run was composed of 10 trials with a 9 s ITI, and thus a total of 150 trials were completed. Each run included a 12 s rest period before the first trial and another 12 s rest period after the last trial. In total, each run lasted for 232 s and 116 functional volumes were collected per run.

#### 2.2.6. Behavioral Analysis

To evaluate the task performance, we calculated the number of correct sequences completed within the 10 s execution period in each trial [6]. We also identified the timing when a movement error occurred (i.e., pressing incorrect button) in each trial. We used the number of correct sequences and the timing of the error occurrence in the following fMRI data analysis.

It is well known that a motor performance tends to be worse at the beginning of an experimental session even when a motor task is well trained (warm-up decrement) [8, 9]. Thus, to confirm whether the task was well trained and no significant learning effect occurred during the experiment, we calculated the mean number of correct sequences across 10 trials in the 3rd and the last (15th) runs separately in each participant and compared them using a paired *t*-test. To check the warm-up decrement effect, we also calculated the mean number of correct sequences across 20 trials performed during the 1st and 2nd runs and during the 3rd and 4th runs separately in each participant and compared them.

#### 2.2.7. fMRI Data Analysis

In the preprocessing of the functional volumes, we initially performed slice timing correction and head motion correction (realignment). After these corrections, both the functional and anatomical images were normalized to the Montreal Neurological Institute (MNI) template brain using the standard SPM8 defaults. The functional images were smoothed with an isotropic 8 mm full-width-at-half-maximum Gaussian kernel. Finally, high-pass filters (128 s) were applied to the fMRI time series in each run to remove low frequency noise and global changes in the signals.

The statistical analysis was performed on two levels. A first-level analysis was done in each participant as follows. A linear regression model (general linear model) was fitted to the data obtained from each participant. The model included the following three regressors: (1) a performance-related regressor (PERFORMANCE), which was a parametric modulation regressor for the number of correct sequences in each trial; (2) an error-related regressor (ERROR), where each timing of incorrect button press was modeled as the event-related regressor [10]; and (3) a task-related regressor (TASK), where each 10 s execution period was simply modeled with a boxcar function. These regressors were convolved with the canonical hemodynamic response function (HRF) in SPM8. We also included six head motion parameters estimated in the realignment procedure as regressors in each session to minimize the effects of head motion artifacts. Then, we generated a contrast image for each of three regressors, that is, the PERFORMANCE, ERROR, and TASK images. By doing so, we depicted brain regions where the activity changed in association with the fluctuation of the task performance (PERFORMANCE image), brain regions where activity was related to the incorrect response (ERROR image), and brain regions active during the executing of the motor task (TASK image).

To accommodate interparticipant variability, each participant's contrast image was entered into a random-effect group analysis [11]. In this analysis, one-sample *t*-test was used. We adopted a voxelwise threshold of *p* < 0.001 (uncorrected) and evaluated significance of brain activation in terms of spatial extent of the activation in the entire brain (*p* < 0.05 familywise error [FWE] corrected for multiple comparisons). For anatomical identification of the significant clusters of the voxels, we referred to the SPM Anatomy toolbox (version 1.8) [12].

### 2.3. tDCS Experiment

In the fMRI experiment, we identified the clusters of active voxels in the bilateral DLPFC, bilateral frontoparietal regions, and the left cerebellum in the PERFORMANCE image (see Section 3). This suggested that the activity in these regions changed in association with the fluctuation of the task performance (= number of correct sequences). Then, in the next tDCS experiment, we tried to modulate brain activity in the right DLPFC with tDCS and tested if the tDCS could affect the task performance. We selected the DLPFC because it is generally believed that the DLPFC is the highest cortical area that is involved in motor planning.

#### 2.3.1. tDCS Settings

To stimulate the right DLPFC, the target electrode was placed on F4 according to the EEG international 10-20 system [13, 14]. The reference electrode was placed in the area of the contralateral supraorbital region. Special care was taken to place the reference electrode more than 5 cm away from the target electrode [15].

A 15 min tDCS with 2 mA was applied from an electrical stimulator (DC-stimulator-Pulse M, neuroConn, Germany) via two saline-soaked surface sponge electrodes (5 × 7 cm). We used three types of stimulation: anodal (anodal electrode on F4), cathodal (cathodal electrode on F4), and sham. Sham stimulation was comprised of a short-period (30 s) current stimulation with the same polarity as the anodal stimulation. A fade-in and fade-out period was set at 30 s at the beginning and at the end of stimulation. In the sham session, we did not inform the participants of this (single blind).

#### 2.3.2. Experimental Procedure

Each participant completed three sessions (anodal, cathodal, or sham session) on separate days. The order of the stimulation was randomized. Each session was conducted at least 7 days apart in order to minimize the risk of contamination via the carryover effects from the previous tDCS application. In the tDCS experiment, the participants sat comfortably in a chair with the right forearm on an armrest, allowing them to relax their arms. Beneath their hands, the same button device used in the fMRI experiment was placed and the same visual instructions as in the fMRI experiment were presented on a computer monitor in front of the participant (Figure 1(b)). All participants completed a session of 4 experimental runs. As in the fMRI experiment, each run consisted of 10 trials with 9 s ITI and included a 12 s rest period before the first trial and after the last trial (232 s).

In the first two runs, the participants performed the task without receiving any stimulation (1st and 2nd runs; PRE). These were done to measure the baseline level of their performance for a session. Then, they performed the task by receiving either type (anodal, cathodal, or sham) of the tDCS to the right DLPFC in the next two runs (3rd and 4th runs; DURING). We started the tDCS immediately after the 2nd run was completed and the stimulation lasted until the end of the 4th run in the anodal and cathodal sessions. We set a 5 min interrun interval between the 2nd and the 3rd runs, because it is shown that neuronal modulation effect by tDCS may emerge around 5 min after the initiation of the stimulation [14, 16]. We set a 2 min interrun interval between the 1st and the 2nd runs and between the 3rd and the 4th runs.

#### 2.3.3. tDCS Data Analysis

*Evaluation of Task Performance*. We calculated the mean number of correct sequences across 20 trials performed in the 1st and the 2nd runs (PRE) and in the 3rd and the 4th runs (DURING) separately. These were calculated for each session (anodal, cathodal, or sham) in each participant separately. We then assessed the tDCS effect by subtracting the mean obtained in the PRE from that obtained in the DURING in each session and calculated the mean tDCS effect across participants for each session. Statistical evaluation was done using one-way repeated-measures analyses of variance (_RM_ANOVA) with a within-subject factor of session (anodal, cathodal, and sham). In this analysis, since Mauchly's test indicated that the sphericity assumption was violated, the Greenhouse-Geisser correction coefficient epsilon was used for correcting the degrees of freedom.

*Evaluation of Performance Variability.* We also calculated the standard deviation (SD) of the number of correct sequences across 20 trials performed in the 1st and the 2nd runs (PRE) and in the 3rd and the 4th runs (DURING) separately. We first calculated these for each session (anodal, cathodal, or sham) in each participant separately and calculated the mean tDCS effect across participants for each session.

## 3. Results and Discussion

### 3.1. Behavioral Results in the fMRI Experiment

**Figure 2:**
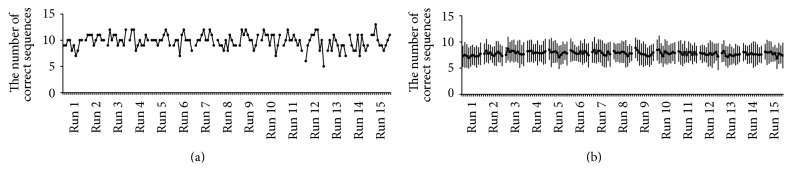
(a) Trial-by-trial fluctuation in the number of correct sequences in a representative participant. (b) Mean number of correct sequences across all 15 participants. The error bar indicates ±1 SD.

When we looked at the change in the number of correct sequences (= task performance) in each participant, we found that the task performance fluctuated trial by trial even though no gradual performance improvement was observed throughout the experimental runs (Figure 2(a)). Indeed, the mean number of correct sequences across participants was stable throughout the experimental runs (Figure 2(b)). We confirmed that the performance in the last (15th) run was not significantly different from that in the 3rd run (df = 14; *p* > 0.05, *d* = 0.14), suggesting that no significant learning effect occurred during the experiment.

Despite this stable performance, we found warm-up decrement effect in the performance of the 1st and 2nd runs. Namely, the mean number of correct sequences in the 1st and 2nd runs (7.56 ± 1.76; mean ± SD) across participants was significantly smaller than that in the 3rd and 4th runs (8.01 ± 1.83; *p* < 0.05, *d* = 0.25). We do not think that this may rebuff our view that no significant learning effect occurred during the experiment, because it is known that a motor performance tends to be worse at the beginning of an experimental session even when a motor task is well trained [8, 9].

These lines of evidence suggested that even though the participants well trained the task and the performance was stable during the experiment, the performance could still fluctuate trial by trial.

### 3.2. fMRI Results

**Figure 3:**
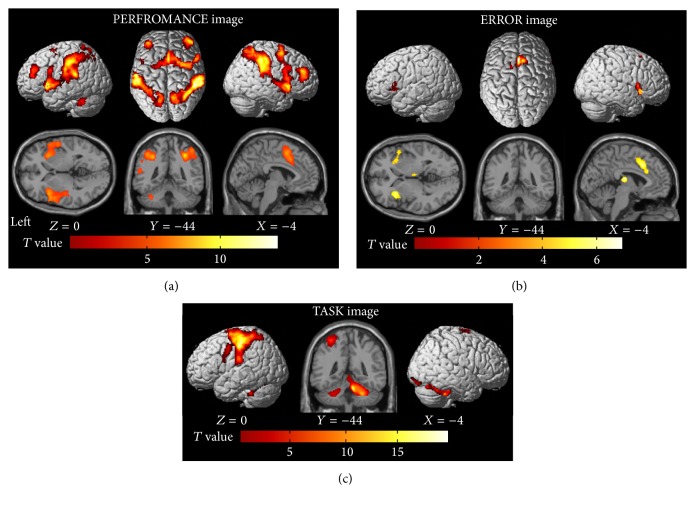
(a) Brain regions where activity was negatively correlated with the number of correct sequences (PERFROMANCE image). (b) Brain regions where activity was related to the occurrence of movement errors (ERROR image). (c) Brain regions active during the 10 s execution period (TASK image). Activities are superimposed on the MNI standard brain.

When we analyzed the PERFORMANCE image, we found that activity in a broader range of brain regions was negatively correlated with task performance across trials (Figure 3(a)). These brain regions included the bilateral DLPFC, pre-supplementary motor area (pre-SMA), middle cingulate cortex, the right premotor cortex, insular cortex, superior and inferior parietal lobules, precuneus, supramarginal gyrus, and the left cerebellum (Table 1). This means that the deterioration in the task performance among trials was associated with the greater activity in the frontoparietocerebellar network.

It should be noted that the task performance was negatively correlated with the number of movement errors (mean of *r* = −0.66 ± 0.14, range from −0.4 to −0.87, *p* < 0.01 for all participants). Thus, it was likely that the greater frontoparietocerebellar activity (Figure 3(a)) contains transiently augmented activity associated with the occurrence of movement errors. Indeed, when we analyzed the ERROR image, we found that the activity in the bilateral pre-SMA, anterior and middle cingulate cortices, insular cortices, and thalamus increased at the timing of error occurrence (Figure 3(b) and Table 2). Since the cingulate and insular cortices were also depicted in the PERFORMANCE image (Figure 3(a)) and these brain regions are known to increase their activity when a movement error occurs [17, 18], we assume that their augmented activities were most likely attributed to error detection and/or awareness [19, 20].

On the other hand, it seems that the greater activity in the bilateral DLPFC, superior and inferior parietal lobules, premotor cortices, and left cerebellum may not reflect such error-related activity (Figures 3(a) and 3(b)). In addition, some of these brain regions including the bilateral DLPFC and the posterior parts of the parietal lobules were not significantly activated during the execution of the task (TASK image, Figure 3(c) and Table 3). This indicates that these brain regions are not directly involved in the execution of the motor task per se. We are speculating that the activity in these regions likely reflects the endogenous fluctuation of neuronal activity in the brain, and we may attribute the trial-by-trial performance fluctuation in the well-trained motor task to the fluctuation of brain activity in the frontoparietocerebellar network (Figure 3(a)).

The fact that this network included the premotor cortex nicely supported the previous nonhuman primate's finding [2]. In addition, the new finding that the regions showing the activity fluctuation belong to the nonmotor domains in the brain, which are not directly involved in the execution of the motor task, supported our view that neuronal fluctuation in the nonmotor domains may also be a cause of fluctuation in a motor performance.

### 3.3. tDCS Results

**Figure 4:**
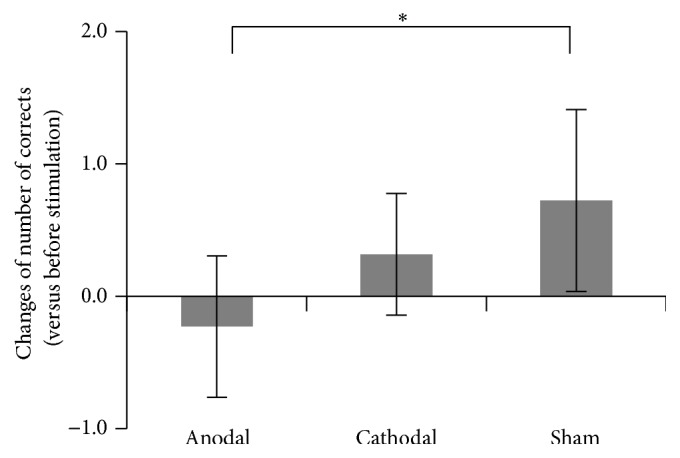
Mean tDCS effect on the number of correct sequences across participants in each (anodal, cathodal, or sham) session. The error bar indicates ±1 SD. ^*∗*^ *p* = 0.019 (uncorrected).

When we evaluated the tDCS effect on the number of correct sequences, we found small performance improvement in the DURING (3rd and 4th runs) of the sham session (0.72 ± 0.69, Figure 4). The subtle improvement was also observed in the cathodal session (0.32 ± 0.46); however, the performance was deteriorated in the anodal session (−0.22 ± 0.53, Figure 4). It can be said that the deterioration effect observed in the anodal session was prominent when compared to the case where the performance was improved in the sham session, since the deterioration effect observed in the anodal session as compared to the improvement effect in the sham session was found in eight out of nine participants. Indeed, the _RM_ANOVA showed a significant main effect of the session (*F*(2,16) = 5.54, *p* < 0.05, *η*_*p*_^2^ = 0.41), and post hoc *t*-test showed a significant difference in the tDCS effect between anodal and sham sessions (df = 8; *p* = 0.019 uncorrected, *d* = 1.54). No such difference was observed in the other two comparisons (between sham and cathodal: *p* = 0.13 uncorrected, *d* = 0.70, anodal and cathodal: *p* = 0.09 uncorrected, *d* = 1.09).

On the contrary, when we evaluated the tDCS effect on the standard deviation (SD) of the number of correct sequences (= degree of performance variability), we could not find any significant change between the PRE and the DURING in any of the tDCS sessions (anodal: PRE 1.33 ± 0.62, DURING 1.40 ± 0.53; cathodal: PRE 1.42 ± 0.60, DURING 1.38 ± 0.69; sham: PRE 1.30 ± 0.45, DURING 1.31 ± 0.47) nor in the change of SD in the DURING as compared to the PRE across three tDCS sessions. Hence, the degree of performance variability per se did not change across three tDCS (anodal, cathodal, or sham) sessions, while the anodal tDCS deteriorated the task performance when compared with the sham.

We should carefully discuss the performance improvement in the sham session (Figure 4). This means that the performance was better in the DURING (3rd and 4th runs) when compared with the PRE (1st and 2nd runs), which in turn means that the performance was worse in the 1st and 2nd runs. This seems to nicely fit to the behavioral finding in the fMRI experiment where we found the warm-up decrement effect in these early runs. Thus, we may attribute the performance improvement in the DURING of the sham session to the warm-up decrement effect.

If the anodal tDCS did not give any impacts to the brain activity, we could have expected the performance improvement also in the anodal session. But the results in the anodal session showed the opposite and significantly different effect from the sham session. This seems to corroborate our view that the present anodal tDCS gave significant impacts to the brain activity and that this tDCS effect likely deteriorated the task performance without affecting the degree of performance variability.

In the present study, we showed the possible causal relationship between the anodal tDCS to the right DLPFC and the task performance. However, we could not elucidate exact neuronal mechanisms of how the tDCS modulated brain activity so as to affect the motor performance. One possibility is that anodal tDCS modulated local brain activity in the right DLPFC. Previous neurophysiological studies demonstrated that anodal tDCS may provide a potentiation effect on neuronal excitability in a stimulated brain region [16, 21, 22]. Thus, theoretically the neuronal excitability in the right DLPFC can be potentiated by its anodal stimulation [14]. If this view is correct, we may speculate that anodal tDCS increased the neuronal excitability in the DLPFC and its artificially potentiated brain activity would increase the likelihood of neuronal fluctuation in the DLPFC so as to deteriorate the task performance.

Another possibility is that anodal tDCS could affect the activity in the remote nonstimulated brain regions that are functionally and anatomically connected with the DLPFC. A previous study showed that anodal tDCS to the right DLPFC can modulate the activity not only in this region but also in the left DLPFC and in the bilateral posterior parts of the parietal lobules [13]. Anyhow, both views seem to be generally consistent with the present fMRI finding with no tDCS that the greater the brain activity in the nonmotor frontoparietocerebellar network that includes DLPFC, the lower the task performance (Figure 3(a)).

We should also carefully discuss the effect of cathodal tDCS (Figure 4). The performance seems to be slightly improved under the cathodal tDCS, which was not significantly different from the sham effect (= natural warm-up effect). This means that even though the performance slightly improved under the cathodal tDCS, this effect was not more than the effect of sham stimulation, which should not give substantial influence on the brain activity. Thus, it might be safe to say that the cathodal tDCS did not give substantial influence on the brain activity so as to produce additional behavioral improvement compared to the sham, even though the original effect of the cathodal tDCS has been considered to decrease neuronal excitability in a stimulated region [16, 23].

The effect of cathodal stimulation is still controversial [24] and probably depends on stimulation parameters (e.g., intensity or durations). Indeed, a recent study shows that 2 mA cathodal tDCS may increase the cortical excitability in the primary motor cortex (M1) [25].

The reason why we could not find stronger improvement effect in the cathodal session than in the sham session could also be explained by ceiling effect. In general, when a motor task is well trained, its further performance improvement is normally difficult to achieve. Indeed, it is shown that facilitative tDCS to the M1 only improves the performance of a motor skill in novices but not in experts [26, 27].

Taken together, we showed in the tDCS experiment that neuromodulation with anodal tDCS to the DLPFC (the representative nonmotor region where activity showing neuronal fluctuation associated with the performance fluctuation in the fMRI experiment) may affect the task performance without affecting the degree of performance variability per se, though we could not elucidate exact neuronal mechanisms underlying the tDCS effect.

## 4. Conclusion

The present study aimed to investigate a distributed neuronal cause for trial-by-trial fluctuation in well-learned skillful motor performance. We showed that the fluctuation of brain activity in the nonmotor frontoparietocerebellar network is associated with the trial-by-trial performance fluctuation even in a well-learned skillful motor task and that neuromodulation with anodal tDCS to the representative nonmotor domain (DLPFC) may affect the task performance.

## Figures and Tables

**Table 1:** Brain regions where activity was negatively correlated with the number of correct sequences (PERFROMANCE image).

Brain areas	Coordinates of peaks			*Z*-value
	*x*	*y*	*z*	
Right hemisphere				
MFG	36	44	26	4.52
IFG	36	32	26	4.00
MCC	10	24	38	4.65
45	56	20	30	4.28
6	10	12	56	4.91
Insula	38	10	−10	4.76
PF	62	−32	28	4.90
PFt	50	−36	48	4.84
hiP2	40	−34	38	4.38
7PC	42	−48	58	4.46
7A	34	−56	54	5.48

Left hemisphere				
MFG	−34	40	16	4.26
MCC	−10	18	36	4.91
44	−46	10	26	4.36
6	−4	8	52	4.49
Insula	−42	16	−2	4.67
STG	−50	4	−2	4.23
PG	−62	−18	40	4.28
2	−52	−24	38	4.90
PF	−62	−36	20	4.89
PFt	−60	−16	30	4.83
hiP2	−40	−44	48	4.41
hiP3	−30	−50	44	6.04
Lobule VI	−32	−50	−36	4.67
Lobule VII	−44	−52	−34	4.42

Peaks in brain activation that were more than 4 mm apart from each other were reported (voxel size = 2 × 2 × 2 mm). For anatomical identification of peaks, we only considered cytoarchitectonic areas with more than 30% probability available in the anatomy toolbox. Cytoarchitectonic area with the highest probability was reported for each peak. When cytoarchitectonic areas with more than 30% probability were not available for a peak, we simply provided its anatomical location. When several peaks were identified at the same cytoarchitectonic area or anatomical location, we only provided the peak coordinates with the highest *Z*-value. MFG: middle frontal gyrus. IFG: inferior frontal gyrus. MCC: middle cingulate cortex. STG: superior temporal gyrus. PG: postcentral gyrus.

**Table 2:** Brain regions where activity was related to the occurrence of movement errors (ERROR image).

Brain areas	Coordinates of peaks			*Z*-value
	*x*	*y*	*z*	
Right hemisphere				
ACC	6	32	28	3.53
MCC	10	20	42	4.16
IFG	52	14	−2	3.51
44	56	12	8	3.69
6	8	18	58	3.96
Insula	40	18	−4	3.70
Thalamus	12	−14	12	3.52

Left hemisphere				
ACC	−4	26	28	3.76
MCC	−8	20	38	4.01
44	−38	20	12	3.78
SFG	−14	12	48	3.67
Insula	−32	20	4	4.37
SMG	−2	18	42	4.19
Thalamus	−4	−18	12	4.44

For anatomical identification of peaks, we used the same criterion adopted in Table 1. ACC: anterior cingulate cortex. MCC: middle cingulate cortex. IFG: inferior frontal gyrus. SFG: superior frontal gyrus. SMG: superior medial gyrus.

**Table 3:** Brain regions active during execution period (TASK image).

Brain areas	Coordinates of peaks			*Z*-value
	*x*	*y*	*z*	
Right hemisphere				
6	2	−6	70	4.29
LG	14	−90	−14	3.60
18	22	−86	−16	3.41
17	22	−94	−16	3.40
Vermis	6	−64	−26	6.28
Lobule V	2	−56	−2	5.03
Lobule VI	22	−56	−26	6.77
Lobule VIIa	40	−72	−24	4.13

Left hemisphere				
SFG	−22	−8	56	4.53
6	−40	−18	64	4.98
4a	−34	−24	56	5.37
1	−54	−20	48	5.41
2	−38	−30	46	5.66
PFop	−56	−20	28	5.05
Lobule VI	−20	−62	−24	4.95

For anatomical identification of peaks, we used the same criterion adopted in Table 1. SFC: superior frontal gyrus. LG: lingual gyrus.
